# TNFAIP8 variants as potential epidemiological and predictive biomarkers in ovarian cancer

**DOI:** 10.1186/s12935-020-01490-7

**Published:** 2020-08-17

**Authors:** Hongyu Gao, Zhiran Zhang, Liangliang Jiang, Lei Zhang, Ling Qin, Tianbo Liu, Shanshan Yang

**Affiliations:** 1 Department of Gastroenterologic Surgery, Harbin Medical University Cancer Hospital, Harbin Medical University, Harbin, 150081 China; 2 Department of Gynecology, Harbin Medical University Cancer Hospital, Harbin Medical University, 150 Haping Road, Harbin, 150081 China; 3 Department of Ultrasound, Harbin Medical University Cancer Hospital, Harbin Medical University, Harbin, 150081 China; 4 Department of Pathology, Harbin Medical University Cancer Hospital, Harbin Medical University, Harbin, 150081 China; 5 Department of Gynecological Radiotherapy, Harbin Medical University Cancer Hospital, Harbin Medical University, 150 Haping Road, Harbin, 150081 China

**Keywords:** TNFAIP8 polymorphism, Ovarian cancer, Susceptibility, rs11064, rs1045242, Predictive biomarkers, Recurrence

## Abstract

**Background:**

This research aimed to investigate the association between tumor necrosis factor-a-induced protein 8 (TNFAIP8) polymorphisms and ovarian cancer (OC) susceptibility.

**Methods:**

A case–control study of 210 patients with OC and 231 healthy controls was conducted to assess the association between TNFAIP8 polymorphisms (rs11064, rs1045241, and rs1045242) and OC risk in Heilongjiang Province of China. The SNaPshot SNP assay was conducted to detect SNP genotype. Logistic regression analysis was applied to illustrate the underlying association.

**Results:**

Our research found that TNFAIP8 rs11064 and rs1045242 were significantly connected with the susceptibility of OC. Additionally, rs1045242 increased the risk of OC, while rs11064 performed a protective role in the risk of OC. Data revealed that rs1045242 strongly related with advanced FIGO stage, larger residual tumor, and the presence of recurrence.

**Conclusions:**

TNFAIP8 genetic variants, which may play difference roles, were significantly associated with OC susceptibility. The underlying molecular mechanism needs be clarified with scientific evidence.

## Background

More than 3000 women a year were diagnosed ovarian cancer (OC) and two third of them ultimately die in the next 5 years [1, 2]. Furthermore, the incidence and mortality of Chinese women with OC has increased significantly [3]. However, no worthily diagnostic methods worldwide were applied for early detection of OC resulting in that OC were more common in advanced clinical stages [2]. Regarding that OC is a multigenic disease [4, 5], the influence of environmental on its pathogenesis should not be neglected [6]. Therefore, it may be an interesting option to investigate key genes and their interaction with the environment for prevention and treatment of OC.

Tumor necrosis factor-a-induced protein 8 (TNFAIP8), as well as a TNFα-inducible gene in endothelial cells [7], was localized at chromosome 5 in the forward strand q23 region [8, 9]. TNFAIP8 takes part in the process of apoptosis and autophagy in different types of cells. Overexpression of TNFAIP8 is frequently observed in malignant tumors [8, 10–20], that is significantly correlated to excessive proliferation, reduced apoptosis, enhanced invasion and metastasis, and drug resistance. Polymorphisms of TNFAIP8 gene are reported to be associated with risks of different cancers [9, 14, 21]. Additionally, we have demonstrated that elevated expression of TNFAIP8 protein implies poor prognosis and is related with resistance of OC [13, 22, 23]. However, there were no existing findings regarding the relationship of TNFAIP8 polymorphisms with OC risks. Therefore, we aimed to clarify the connection between TNFAIP8 polymorphism and OC susceptibility among people in Heilongjiang Province of China.

## Materials and methods

### Subjects and blood samples

Totally 210 OC patients and 231 contemporaneously healthy individuals were recruited from the Harbin Medical University Cancer Hospital between September 2015 and February 2017. All OC cases were classified and evaluated according to the International Federation of Obstetricians and Gynecologists (FIGO) [24]. The pathological type was diagnosed as epithelial OC which contained serous, mucinous, endometrioid, and clear cell histological type. Exclusion criteria: (1) Any of the recruited patients who received preoperative chemo-, radio- or immunotherapy; (2) any control subject with malignant tumor or digestive disease, or the family history of any cancers; (3) incomplete clinical case data or incomplete follow-up information. Peripheral blood samples (5 mL) were collected from all subjects at the time of hospital admission.

The distributions of clinical data of all subjects are shown in Table 1. The study protocol was approved by Harbin Medical University Cancer Hospital Committee (ethical number: KY2019-09) and all subjects provided signed informed consent from patients and controls.

**Table 1:** Demographic and clinicopathologic characteristics of 210 ovarian cancer cases and 231 healthy controls

Characteristics	Cases	Controls	P*
Age	53.24 ± 10.54	54.32 ± 9.58	0.261
BMI	25.40 ± 3.65	25.47 ± 3.52	0.814
Family cancer history (ovarian cancer)			0.023
No	203	230	
Yes	7	1	
Parity			0.415
Nulliparity	32	29	
Multiparity	178	202	
Complication^a^			0.060
No	158	155	
Yes	52	76	
Smoking history			0.583
No	159	180	
Yes	51	51	
FIGO stage			
I/II	94		
III/IV	116		
Histologic grade			
G1/G2	95		
G3	115		
Histological type			
Serous	132		
Mucinous	31		
Endometrioid	32		
Clear cell	15		
Residual tumor size			
≤ 1 cm	132		
> 1 cm	78		
Ascites			
≤ 100 ml	64		
> 100 ml	146		
Serum CA-125 level			
≤ 35 U/ml	45		
> 35 U/ml	165		
Recurrence			
No	124		
Yes	86		

*BMI* body mass index, *FIGO* the Federation of Gynaecology and Obstetrics, *G1* Well differentiated, *G2* moderately differentiated, *G3* poorly differentiated

* Two-sided Chi squared test or Fisher’ s text or student’s t text

^a^Complication: patients with diabetes and cardio-cerebrovascular disease

### Genotyping

Peripheral blood (5 ml) from each subject was sampled in vacuum tubes with 5% ethylene diamine tetraacetic acid (EDTA). Then genomic DNA from whole blood was extracted using a blood genomic DNA extraction kit (Axygen Biotechnology, Union City, CA, USA) according to the manufacturer’s instruction and stored at − 20 °C for genotyping by polymerase chain reaction (PCR). Three TNFAIP8 SNPs (rs11064, rs1045241, and rs1045242) were selected in the present study according to our previous study [21], and we used Primer Blast to design the PCR amplification primers as follows: The PCR mixture contained 100 ng of genomic DNA, 4 μl of 2.5 mM dNTP, 10 μl of PCR buffer, 10 μM of upstream and downstream primers, 1 μl each, 0.5 U of PrimeSTAR HS DNA polymerase (TAKARA, DALIAN, China) in a 50-μl reaction volume. The PCR amplification conditions were: 94 °C, 5 min, 35 cycles; 98 °C, 10 s; 58 °C, 15 s; 72 °C, 2 min, final extension step, 72 °C, 5 min. Then, the SNaPshot SNP assay was conducted to detect SNP genotype. The GeneMapperTM 4.0 Software (Applied Biosystems, Foster City, CA, USA) was applied to analyzed the resulting data. About 5% of the specimens were chosen randomly and genotyped twice to ensure the genotyping accuracy: the reproducibility was 100% [21].

### Statistical analysis

All statistical analyses were performed with SPSS 22.0 (SPSS, Chicago, Illinois, USA). Genotype and allele distributions were assessed and the chi-square test was used to evaluated the Hardy–Weinberg equilibrium among the controls. Continuous variables were presented using mean ± SD and statistically analyzed using t-test. Categorical variables were statistically analyzed using the chi-square test or Fisher’s text. The crude odds ratio (COR), adjusted odds ratio (AOR), and 95% confidence interval (CI) of logistic regression analysis was calculated in four genetic models (allele, co-dominant, dominant, and recessive) to assess the association between TNFAIP8 single nucleotide polymorphisms (SNPs) and OC susceptibility with adjustment for age, smoking history, complication, and family history. All tests were two-tailed and *P* < 0.05 was considered statistical significance.

## Results

### Demographic characteristics the of study population

The connection between TNFAIP8 SNPs and OC risk was explored in Heilongjiang Province of China. The basic information of all individuals was summarized in Table 1. The average ages of cases and controls were 53.24 ± 10.54 and 54.32 ± 9.58 years, respectively. Furthermore, no significant difference was observed between these two groups (*P* = 0.261). Also, there was no significant difference of body mass index (BMI) between two groups (*P* = 0.814). In addition, there were no significant differences between the cases and controls in Parity, complication and smoking history (*P* > 0.05). However, positive significance (*P *= 0.023) between the case and control groups was presented in family cancer history (ovarian cancer).

### The relationship of TNFAIP8 polymorphism with OC risk

In this case–control study, three SNPs (rs11064, rs1045241, and rs1045242) which are located in the 3′ UTR, which is a binding site for the regulation of gene expression by microRNAs (miRNAs) in TNFAIP8 gene were selected and analyzed [21]. The genotype frequencies of each SNP conformed to the Hardy–Weinberg equilibrium among controls (*P* > 0.05 for all). Displayed in Table 2, TNFAIP8 rs11064 A-allele (COR: 0.690, 95% CI 0.491–0.971, *P *= 0.033 and AOR: 0.709, 95% CI 0.504–0.997, *P *= 0.048) and rs1045242 G-allele (COR: 1.619, 95% CI 1.129–2.323, *P *= 0.009 and AOR: 1.628, 95% CI 1.132–2.342, *P *= 0.009) are risk factors for OC. However, the allele of TNFAIP8 rs1045241 had no effect on the risk of OC (*P* > 0.05).

**Table 2:** The distribution of allele frequencies of TNFAIP8 SNPs in cases and controls

Variables	Cases (%) n = 420	Controls (%) n = 462	COR (95% CI)	*P*	AOR (95% CI)	P*
rs11064						
A	352 (83.8)	361 (78.1)	1.000		1.000	
G	68 (16.2)	101 (21.9)	0.690 (0.491–0.971)	*0.033*	0.709 (0.504–0.997)	*0.048*
rs1045241						
C	341 (81.2)	372 (80.5)	1.000		1.000	
T	79 (18.8)	90 (19.5)	0.958 (0.684–1.340)	0.800	0.960 (0.684–1.349)	0.816
rs1045242						
A	337 (80.2)	401 (86.8)	1.000		1.000	
G	83 (19.8)	61 (13.2)	1.619 (1.129–2.323)	*0.009*	1.628 (1.132–2.342)	*0.009*

*COR* crude odds ratio, *AOR* adjusted odds ratio, *CI* confidence interval

* Data were calculated by logistic regression, adjusted for age, smoking history, complication, family history

For further examination, we conducted the correlation between the genotypes of SNPs and OC risk by logistic regression analysis under the codominant, dominant, and recessive models (Table 3). Our results showed that rs11064 was significantly associated with increased OC susceptibility in codominant model (GG/AA, COR: 0.200, 95% CI 0.057–0.706, *P *= 0.012 and AOR: 0.205, 95% CI 0.058–0.726, *P *= 0.014) and recessive model (GG/AA + AG, COR: 0.209, 95% CI 0.060–0.731, *P *= 0.014 and AOR: 0.212, 95% CI 0.060–0.744, *P *= 0.016). Also, we demonstrated that rs1045242 was related to a higher risk of OC under codominant model (AG/AA, COR: 1.670, 95% CI 1.091–2.558, *P *= 0.018 and AOR: 1.703, 95% CI 1.108–2.618, *P *= 0.015) and dominant model (AG + GG/AA, COR: 1.736, 95% CI 1.149–2.623, *P *= 0.009 and AOR: 1.761, 95% CI 1.162–2.670, *P *= 0.008). However, there was no significant association between TNFAIP8 rs1045241 and OC risk.

**Table 3:** Relationship of TNFAIP8 polymorphisms and ovarian cancer risk

Variables	Cases (%) n = 210	Controls (%) n = 231	COR (95% CI)	*P*	AOR (95% CI)	P*
rs11064						
Codominant						
AA	145 (69.0)	145 (62.8)	1.000	*0.040*	1.000	*0.048*
AG	62 (29.5)	15 (6.5)	0.873 (0.579–1.317)	0.518	0.905 (0.598–1.370)	0.636
GG	3 (1.4)		0.200 (0.057–0.706)	*0.012*	0.205 (0.058–0.726)	*0.014*
Dominant						
AA	145 (69.0)	145 (62.8)	1.000		1.000	
AG + GG	65 (31.0)	86 (37.2)	0.756 (0.509–1.123)	0.166	0.782 (0.524–1.165)	0.226
Recessive						
AA + AG	207 (98.6)	216 (93.5)	1.000		1.000	
GG	3 (1.4)	15 (6.5)	0.209 (0.060–0.731)	*0.014*	0.212 (0.060–0.744)	*0.016*
rs1045241						
Codominant						
CC	137 (65.2)	154 (66.7)	1.000	0.276	1.000	0.214
CT	67 (31.9)	64 (27.7)	1.177 (0.779–1.778)	0.440	1.216 (0.801–1.846)	0.359
TT	6 (2.9)	13 (5.6)	0.519 (0.192–1.402)	0.196	0.497 (0.183–1.350)	0.170
Dominant						
CC	137 (65.2)	154 (66.7)	1.000		1.000	
CT + TT	73 (34.8)	77 (33.3)	1.066 (0.718–1.581)	0.752	1.089 (0.731–1.622)	0.674
Recessive						
CC + CT	204 (97.1)	218 (94.4)	1.000		1.000	
TT	6 (2.9)	13 (5.6)	0.493 (0.184–1.322)	0.160	0.468 (0.174–1.263)	0.134
rs1045242						
Codominant						
AA	135 (64.3)	175 (75.8)	1.000	*0.026*	1.000	*0.025*
AG	67 (31.9)	52 (22.5)	1.670 (1.091–2.558)	*0.018*	1.703 (1.108–2.618)	*0.015*
GG	8 (3.8)	4 (1.7)	2.593 (0.765–8.791)	0.126	2.490 (0.731–8.484)	0.145
Dominant						
AA	135 (64.3)	175 (75.8)	1.000		1.000	
AG + GG	75 (35.7)	56 (24.2)	1.736 (1.149–2.623)	*0.009*	1.761 (1.162–2.670)	*0.008*
Recessive						
AA + AG	202 (96.2)	227 (98.3)	1.000		1.000	
GG	8 (3.8)	4 (1.7)	2.248 (0.667–7.576)	0.191	2.151 (0.635–7.286)	0.218

*COR* crude odds ratio, *AOR* adjusted odds ratio, *CI* confidence interval

* Data were calculated by logistic regression, adjusted for age, smoking history, complication, family history

### Stratification analysis between TNFAIP8 SNPs and OC risk based on age, smoking history, complication, and family history

Aiming to deeply analyze the relationships of TNFAIP8 genotypes with OC susceptibility, we divided age into ≤ 54 years old and > 54 years old, whether smoking, whether complication (patients with diabetes and cardio-cerebrovascular disease), and whether there is family history of OC. It revealed that rs1045242 mutation (AG + GG/AA) would significantly increase risk of OC (OR: 2.048, 95% CI 1.116–3.757, *P *= 0.021) at age ≤ 54 years old (Additional file 1: Table S1). In subjects with no smoking history, the rs11064 mutation (GG) was a protective factor for OC (OR: 0.164, 95% CI 0.036–0.742, *P *= 0.019). On the contrary, the rs1045242 mutation (AG + GG) was a risk factor for OC (OR: 2.670, 95% CI 1.141–6.247, *P *= 0.024) in subjects with smoking history (Additional file 1: Table S2). As showed in Additional file 1: Tables S3 and S4, the rs1045242 mutation (AG + GG) was a risk factor for OC in subjects with no complication (OR: 1.829, 95% CI 1.109–3.018, *P *= 0.018) and no family history of OC (OR: 1.746, 95% CI 1.150–2.650, *P *= 0.009). The rs11064 GG genotype was a protective factor for OC in subjects with no family history of OC (OR: 0.205, 95% CI 0.058–0.724, *P *= 0.014).

### Correlation between TNFAIP8 SNPs and clinicopathological characteristics of OC

The correlation between three TNFAIP8 genotypes and the clinicopathologic data of OC is illustrated in Table 4. It was found that rs1045242 was related to an increased risk in OC patients with III/IV FIGO stage (*P *= 0.040 and *P *= 0.013, respectively) and presence of recurrence (*P *= 0.043 and *P *= 0.034, respectively) both under codominant and dominant models. For rs1045242, it was confirmed that AG + GG genotype was significantly associated with an increased OC risk in residual tumor more than 1 cm (*P *= 0.019). rs1045241 SNP was strongly significant associated with FIGO stage (*P *= 0.025) and residual tumor (*P *= 0.033) under dominant model. Furthermore, rs11064 SNP was observed to be positively related to FIGO stage both under codominant (*P *= 0.024) and dominant (*P *= 0.006) models.

**Table 4:** The association between TNFAIP8 polymorphisms and clinicopathological characteristics of ovarian cancer

Characteristics	rs11064				P*	rs1045241				P*	rs1045242				P*
	AA	AG	GG	AG + GG		CC	CT	TT	CC + CT		AA	AG	GG	AG + GG	
FIGO stage					*0.024*					0.082					*0.04*
I/II	74	19	1	20	*0.006*	69	23	2	25	*0.025*	69	23	2	25	*0.013*
III/IV	71	43	2	45		68	44	4	48		66	44	6	50	
Histologic grade					0.862					0.827					0.894
G1/G2	67	27	1	28	0.674	63	30	2	32	0.766	62	30	3	33	0.788
G3	78	35	2	37		74	37	4	41		73	37	5	42	
Histological type					–					–					–
Serous	89	40	3	43	0.463	86	40	6	46	0.446	81	40	8	48	0.43
Mucinous	22	9	–	9		21	10	–	10		21	10	–	10	
Endometrioid	21	11	–	11		18	14	–	14		18	14	–	14	
Clear cell	13	2	–	2		12	3	–	3		12	3	–	3	
Residual tumor					0.28					0.098					0.064
≤ 1 cm	86	44	2	46	0.112	79	49	4	53	*0.033*	77	49	6	55	*0.019*
> 1 cm	59	18	1	19		58	18	2	20		58	18	2	20	
Ascites					–					0.739					0.508
≤ 100 ml	46	18	–	18	0.557	43	20	1	21	0.694	43	20	1	21	0.561
> 100 ml	99	44	3	47		94	47	5	52		92	47	7	54	
Serum CA-125					0.800					0.573					0.69
≤ 35 U/m	32	12	1	13	0.736	31	12	2	14	0.562	31	12	2	14	0.467
> 35 U/ml	113	50	2	52		106	55	4	59		104	55	6	61	
Recurrence					0.071					0.112					*0.043*
No	93	30	1	32	0.437	88	33	3	38	0.086	88	33	3	36	*0.034*
Yes	52	32	2	23		49	34	3	35		47	34	5	39	

*FIGO* the Federation of Gynaecology and Obstetrics, *G1* well differentiated, *G2* moderately differentiated, *G3* poorly differentiated

* Two-sided chi-squared test or Fisher’ s text

## Discussion

In present study, we found that TNFAIP8 polymorphisms (rs11064 and rs1045242) were significantly associated with OC susceptibility. Furthermore, the GG-genotype of rs11064 was a protective factor and the AG + GG-genotype of rs1045242 was a risk factor for OC susceptibility. In addition, TNFAIP8 rs1045242 gene polymorphism was linked to advanced FIGO stage, larger residual tumor, and the presence of recurrence in OCs. Taken together, our current findings provided an crucial role of TNFAIP8 gene in the occurrence of OC, thus may give evidence on the potentially functional SNPs in TNFAIP8 and their clinical outcomes in OC patients.

TNFAIP8 polymorphism has been recently investigated in several disease including solid human cancer (cervical cancer and endometrial cancer) and Non-Hodgkin’s Lymphoma (NHL) which indicates that SNPs are the most common type of genetic variations caused by the heterogeneity among various types of human cancer [9, 14, 21]. Recent research suggests that genetic polymorphisms play a crucial role in the pathogenesis of OC [25–27]. To our knowledge, we illuminated the association between TNFAIP8 polymorphism and OC risk for the first time.

In cervical cancer, it revealed that the GG genotype of TNFAIP8 rs11064 was connected with an elevated risk compared with AA/AG genotypes [14]. Furthermore, the study of endometrial cancer (EC) [21] showed that the GG genotype and AG + GG genotype of TNFAIP8 rs11064 were both associated with increased risk compared with controls. However, our present research found that the G allele and GG allele of TNFAIP8 rs11064 both played a reduced role in risk of OC (AOR: 0.709; 95% CI 0.504–0.997 for G allele and AOR: 0.205; 95% CI 0.058–0.726 for GG allele). The discordance of the above findings may be explained by that the effect of genetic factors often differs in different individuals.

No considerable relationship between TNFAIP8 rs1045241 and OC risk was identified in our present paper. Additionally, our previous study in EC had been in accordance with this result [21]. Searching from the literature data, TNFAIP8 rs1045241 polymorphism was reported to have clinical significance in no other reports except that in NHL. Zhang et al. [9] demonstrated that the polymorphism of TNFAIP8 rs1045241 may lead to NHL susceptibility in a Chinese population. We believe that the related role of environmental factors may not be ignored. So far, no literature except our team has reported the relationship between TNFAIP8 rs1045242 polymorphism and tumor. Our results showed that TNFAIP8 rs1045242 G allele carriers showed increased risk of OC by 1.628 times compared to the A allele carriers. Also, the AG + GG genotype of TNFAIP8 rs1045242 increased 1.761 times risks of OC compared with AA genotype. These findings were consistent with previous study in EC [21]. The above provide evidence that TNFAIP8 rs1045242 polymorphism may involve in the onset of gynecological malignancy.

Besides, subgroup analysis revealed that TNFAIP8 rs1045242 polymorphism increased the risk of OC in patients with age ≤ 54 years old, smoking history, no complication, and no family cancer history, uncovering that individuals exposed to these factors are more susceptible to OC. In patients with no smoking history and no family cancer history, the GG allele of TNFAIP8 rs11064 SNPs played a protective factor for OC. However, the underlying mechanism that the same genotype performs opposite effects in different tumor types remains to be illuminated.

Moreover, we explored the connection between the TNFAIP8 genes polymorphism and clinical variables of OC. We suggested that TNFAIP8 genes polymorphism (rs11064, rs1045241, and rs1045242) were significantly connected with FIGO stage. In addition, TNFAIP8 rs1045242 polymorphism was also strongly associated with residual tumor, and recurrence, indicating its role of progression in OC. For rs11064, it was reported that it positively linked to deep myometrial invasion and lymph node metastasis under the codominant model in ECs [21]. In cervical cancer, it attempted to explore the relationship between TNFAIP8 rs11064 polymorphism and drug resistance, but with no sense [14]. The association between TNFAIP8 rs1045242 polymorphism and stage in NHL was observed [9].

The present study is the first to explore TNFAIP8 variants and susceptibility in OC, however, it has come limitations. For example, the follow-up period was not sufficiently long, and our study was retrospective and included a relatively small number of Chinese patients form a single center. Thus, future examination of large sample size and multiple centers are needed to verify genotype–phenotype associations and functional analysis for TNFAIP8 SNP.

## Conclusion

This study suggests that TNFAIP8 rs11064 and rs1045242 polymorphisms are remarkably linked with the risk of OC in Heilongjiang Province of China. However, the GG allele of TNFAIP8 in the two genotypes played the opposite roles in the risk of OC. Furthermore, we found that TNFAIP8 rs1045242 polymorphism had an effect on clinical significance of FIGO stage, residual tumor, and recurrence, indicating its progressive role in OC. Yet, there are some limitations and shortcomings. Whether TNFAIP8 rs1045242 polymorphism affected the protein expression status and its effect on prognosis remain to unclear. It is well-known that the inherited mutations of BRCA1 and BRCA2 genes resulted in hereditary breast and ovarian cancer syndrome (HBOC). However, there are only 7 of 210 OC patients have OC family history and only 1 of 210 OC patients have HBOC in the present case–control study. Thus, well-designed larger including patients with HBOC and hereditary nonpolyposis colon cancer (HNPCC), prospective study with functional analysis is an interesting direction and deserves further study which would give some new insights in the molecular mechanism of OC occurrence.

## Supplementary information

**Additional file 1: Table S1.** Stratified analysis between TNFAIP8 polymorphisms and ovarian cancer risk by age. **Table S2.** Stratified analysis between TNFAIP8 polymorphisms and ovarian cancer risk by smoking history. **Table S3.** Stratified analysis between TNFAIP8 polymorphisms and ovarian cancer risk by complication. **Table S4.** Stratified analysis between TNFAIP8 polymorphisms and ovarian cancer risk by family history.

## Data Availability

All data generated and analyzed during this study are included in this published article and its additional file.
