# Supplementary material for: TNFAIP8 variants as potential epidemiological and predictive biomarkers in ovarian cancer
Source: Cancer Cell Int. 2020 Aug 17;20:396. doi: 10.1186/s12935-020-01490-7 (PMC7433149; doi:10.1186/s12935-020-01490-7)
Supplement: Supplementary file 1 — Additional file 1: Table S1. Stratified analysis between TNFAIP8 polymorphisms and ovarian cancer risk by age. Table S2. Stratified analysis between TNFAIP8 polymorphisms and ovarian cancer risk by smoking history. Table S3. Stratified analysis between TNFAIP8 polymorphisms and ovarian cancer risk by complication. Table S4. Stratified analysis between TNFAIP8 polymorphisms and ovarian cancer risk by family history. [file 12935_2020_1490_MOESM1_ESM.doc]

Table S1. Stratified analysis between TNFAIP8 polymorphisms and ovarian cancer risk by age

|  | Age (years) | | | | | | |
| --- | --- | --- | --- | --- | --- | --- | --- |
|  | ≤ 54 | | |  | > 54 | | |
|  | case/controls | OR (95%CI) | *^*^P* |  | case/controls | OR (95%CI) | *^*^P* |
| rs11064  AA  AG  GG  AG+GG  rs1045241  CC  CT  TT  CT+TT  rs1045242  AA  AG  GG  AG+GG | 79/71  31/34  3/8  34/42  74/76  35/30  4/7  39/37  72/88  35/22  6/3  41/25 | 1.000  0.866 (0.474-1.481)  0.343 (0.085-1.385)  0.764 (0.431-1.353)  1.000  1.254 (0.685-2.296)  0.546 (0.149-1.996)  1.118 (0.632-1.979)  1.000  2.025 (1.068-3.842)  2.195 (0.515-9.365)  2.048 (1.116-3.757) | 0.639  0.133  0.356  0.462  0.360  0.702  0.031  0.288  **0.021** |  | 66/74  31/37  0/7  31/44  63/78  32/34  2/6  34/40  63/87  32/30  2/1  34/31 | 1.000  0.939(0.525-1.680)  -  0.765 (0.430-1.361)  1.000  1.082 (0.594-1.973)  0.477 (0.091-2.491)  0.995 (0.558-1.771)  1.000  1.398 (0.763-2.561)  3.514 (0.307-40.210)  1.456 (0.807-2.627) | 0.833  -  0.362  0.796  0.380  0.985  0.278  0.312  0.212 |

*Data were calculated by logistic regression, adjusted for age, smoking history, complication, family history (excluded the stratified factor in each stratum).

OR: indicates odds ratio, CI: confidence interval.

Table S2. Stratified analysis between TNFAIP8 polymorphisms and ovarian cancer risk by smoking history

|  | Smoking history | | | | | | |
| --- | --- | --- | --- | --- | --- | --- | --- |
|  | No | | |  | Yes | | |
|  | case/controls | OR (95%CI) | *^a^P* |  | case/controls | OR (95%CI) | *^a^P* |
| rs11064  AA  AG  GG  AG+GG  rs1045241  CC  CT  TT  CT+TT  rs1045242  AA  AG  GG  AG+GG | 113/116  44/51  2/13  46/64  109/122  45/49  5/9  50/58  107/136  45/41  7/3  52/44 | 0.897 (0.554-1.454)  0.164 (0.036-0.742)  0.739 (0.466-1.171)  1.035 (0.638-1.679)  0.576 (0.186-1.787)  0.960 (0.606-1.523)  1.399 (0.853-2.295)  2.794 (0.702-11.122)  1.530 (0.949-2.468) | 0.660  **0.019**  0.198  0.890  0.340  0.863  0.183  0.145  0.081 |  | 32/29  18/20  1/2  19/22  28/32  22/15  1/4  23/19  28/39  22/11  1/1  23/12 | 0.864 (0.375-1.989)  0.562 (0.046-6.925)  0.821 (0.366-1.841)  1.676 (0.731-3.842)  0.286 (0.030-2.709)  1.486 (0.663-3.330)  2.786 (1.165-6.659)  1.393 (0.084-23.228)  2.670 (1.141-6.247) | 0.731  0.651  0.632  0.222  0.275  0.336  0.021  0.817  **0.024** |

^a^Data were calculated by logistic regression, adjusted for age, smoking history, complication, family history (excluded the stratified factor in each stratum).

OR: indicates odds ratio; CI: confidence interval.

Table S3. Stratified analysis between TNFAIP8 polymorphisms and ovarian cancer risk by complication

|  | Complication | | | | | | |
| --- | --- | --- | --- | --- | --- | --- | --- |
|  | No | | |  | Yes | | |
|  | case/controls | OR (95%CI) | *^a^P* |  | case/controls | OR (95%CI) | *^a^P* |
| rs11064  AA  AG  GG  AG+GG  rs1045241  CC  CT  TT  CT+TT  rs1045242  AA  AG  GG  AG+GG | 110/99  45/46  3/10  48/56  104/105  48/39  6/11  54/50  102/118  48/34  8/3  56/37 | 0.943 (0.572-1.556)  0.269 (0.071-1.017)  0.818 (0.507-1.321)  1.282 (0.770-2.135)  0.579 (0.204-1.639)  1.128 (0.699-1.819)  1.722 (1.022-2.901)  2.986 (0.763-11.682)  1.829 (1.109-3.018) | 0.819  0.053  0.412  0.339  0.303  0.621  **0.041**  0.116  **0.018** |  | 35/46  17/25  0/5  17/30  33/49  19/25  0/2  19/27  33/57  19/18  0/1  19/19 | 0.907 (0.418-1.968)  -  0.723 (0.336-1.556)  1.096 (0.513-2.339)  -  1.036 (0.488-2.199)  1.775 (0.804-3.917)  -  1.704 (0.777-3.741) | 0.805  -  0.407  0.813  -  0.926  0.156  -  0.184 |

^a^Data were calculated by logistic regression, adjusted for age, smoking history, complication, family history

(excluded the stratified factor in each stratum).

OR: indicates odds ratio; CI: confidence interval.

Table S4. Stratified analysis between TNFAIP8 polymorphisms and ovarian cancer risk by family history

|  | Family history | | | | | | |
| --- | --- | --- | --- | --- | --- | --- | --- |
|  | No | | |  | Yes | | |
|  | case/controls | OR (95%CI) | *^a^P* |  | case/controls | OR (95%CI) | *^a^P* |
| rs11064  AA  AG  GG  AG+GG  rs1045241  CC  CT  TT  CT+TT  rs1045242  AA  AG  GG  AG+GG | 139/144  61/71  3/15  64/86  132/153  65/64  6/13  71/77  130/174  65/52  8/4  73/56 | 0.898 (0.592-1.361)  0.205 (0.058-0.724)  0.775 (0.520-1.157)  1.202 (0.791-1.828)  0.494 (0.182-1.344)  1.077 (0.722-1.606)  1.687 (1.096-2.597)  2.481 (0.728-8.453)  1.746 (1.150-2.650) | 0.611  **0.014**  0.213  0.389  0.167  0.715  **0.018**  0.146  **0.009** |  | 6/1  1/0  0/0  1/0  5/1  2/0  0/0  2/0  5/1  2/0  0/0  2/0 | -  -  -  -  -  -  -  -  - | -  -  -  -  -  -  -  -  - |

^a^Data were calculated by logistic regression, adjusted for age, smoking history, complication, family history

(excluded the stratified factor in each stratum).

OR: indicates odds ratio; CI: confidence interval.
